# Supplementary material for: Prediction of the mechanism of miRNAs in laryngeal squamous cell carcinoma based on the miRNA-mRNA regulatory network
Source: PeerJ. 2021 Aug 24;9:e12075. doi: 10.7717/peerj.12075 (PMC8395572; doi:10.7717/peerj.12075)
Supplement: Supplemental Information 7 [file peerj-09-12075-s007.docx]

Table S1 Differentially expressed miRNAs in LSCC.

| **Differentially expressed miRNAs that are down regulated in LSCC** | **Differentially expressed miRNAs that are up regulated in LSCC** |
| --- | --- |
| hsa-miR-338-3p  hsa-miR-140-5p  hsa-miR-126*  hsa-miR-3654  hsa-miR-218  hsa-miR-3188  hsa-miR-377  hsa-miR-143  hsa-miR-376c  hsa-miR-376a  hsa-miR-136  hsa-miR-1224-5p  hsa-miR-3137  hsa-miR-660  hsa-miR-2276  hsa-miR-497  hsa-miR-186  hsa-miR-30e*  hsa-miR-204  hsa-miR-362-3p  hsa-miR-95  hsa-miR-455-3p  hsa-miR-3682  hsa-miR-582-5p  hsa-miR-590-5p  hsa-miR-140-3p  hsa-miR-10b  hsa-miR-548q  hsa-miR-30a*  hsa-miR-199b-5p  hsa-miR-145*  hsa-miR-455-5p  hsa-miR-3194  hsa-miR-335  hsa-miR-363  hsa-miR-136*  hsa-miR-29c*  hsa-miR-198  hsa-miR-135a  hsa-miR-149*  hsa-miR-532-5p  hsa-miR-3202  hsa-miR-623  hsa-miR-452  hsa-miR-3660  hsa-miR-617  hsa-miR-1972  hsa-miR-3147  hsa-miR-24-1*  hsa-miR-3154  hsa-let-7a  hsa-miR-3919  hsa-miR-126  hsa-miR-221*  hsa-miR-936  hsa-miR-139-5p  hsa-miR-454  hsa-miR-30b*  hsa-miR-4324  hsa-miR-638  hsa-miR-3182  hsa-miR-1180  hsa-miR-194  hsa-let-7f  hsa-miR-16-2*  hsa-miR-29a*  hsa-miR-192  hsa-miR-625  hsa-miR-3185  hsa-miR-4259  hsa-let-7c  hsa-let-7g  hsa-miR-29a  hsa-miR-27b  hsa-miR-3663-5p  hsa-miR-342-5p | hsa-miR-25  hsa-miR-342-3p  hsa-miR-3162  hsa-miR-24  hsa-miR-1305  hsa-miR-1246  hsa-miR-3651  hsa-miR-324-3p  hsa-miR-1274b  hsa-miR-3198  hsa-miR-181a  hsa-miR-1274a  hsa-miR-21*  hsa-miR-1290  hsa-miR-575  hsa-miR-720  hsa-miR-1260  hsa-miR-1280  hsa-miR-193b  hsa-miR-93  hsa-miR-1260b  hsa-miR-92a  hsa-miR-4286 |
