# Supplementary material for: Prediction of the mechanism of miRNAs in laryngeal squamous cell carcinoma based on the miRNA-mRNA regulatory network
Source: PeerJ. 2021 Aug 24;9:e12075. doi: 10.7717/peerj.12075 (PMC8395572; doi:10.7717/peerj.12075)
Supplement: Supplemental Information 8 [file peerj-09-12075-s008.docx]

Table S2 Differentially expressed mRNAs in LSCC.

| **Differentially expressed mRNAs that are up regulated in LSCC** | **Differentially expressed mRNAs that are down regulated in LSCC** |
| --- | --- |
| PIP  STATH  BPIFB1  C6orf58  CRISP2  MYOC  PRR4  SCGB3A1  DMBT1  CRISP3  PRB2  PRH2  ART4  PIGR  GP2  PRB1  FAM3D  ZG16B  OGN  CLCA4  PRB4  C2orf40  CLDN10  AGR3  TCN1  MSMB  SCGB2A1  CYP4B1  AQP5  TFF3  EDN3  KRT4  FMO2  LTF  PLA2G2A  PLP1  CAPN14  TMPRSS11B  CRNN  MAL  CXCL17  GREM2  CHST9  CDH19  PRB3  LPO  FAM3B  LYZ  TFF1  ATP1A2  SH3BGRL2  TSPAN8  LDLRAD1  AZGP1  ATP6V0A4  GABRP  C21orf81  APOD  PSCA  CILP  ANGPTL1  CEACAM5  C21orf15  BPIFB2  C16orf89  SSC5D  CCL14  OXGR1  MIA  SCGB1D1  SCIN  MS4A8B  VIT  PRR15L  ANGPTL7  FAM55B  PADI1  SCRG1  IGF1  SCGB3A2  TMEM132C  LOC100652774  TMEM213  ABCA8  BCAS1  ANKRD20A8P  SORBS2  HMGCS2  KRT13  C2orf54  WFDC2  NME5  PI16  KRT78  BAI3  IL33  GSTA1  RGS22  PTGIS  CHRDL1  GCNT3  ANKRD20A9P  SCN7A  HPGD  C10orf81  TF  PPP1R1B  CLEC3B  MOGAT1  FAM107A  ADH1A  MYRIP  TMC5  NBEA  SPINK5  PLAC8  ST6GALNAC1  NPY1R  SCEL  CRYM  PTGDS  ERBB4  GGTA1P  CYP4X1  MUC5B  CEACAM6  PROM1  GSTA5  CGNL1  ADH7  MMRN1  AGR2  FRMD1  MLPH  PPP1R9A  PEG3-AS1  CACNB4  LOC389791  WISP2  MUC7  TJP3  GDF10  PPP1R3C  RBM20  FOLR1  CYP4Z1  TMEM100  GPR110  ATP12A  VSIG2  CFTR  ANKRD20A5P  SNX31  RSPO1  SRPX  C16orf78  AMPD1  LYVE1  ABI3BP  ABCA9  SFRP2  AR  AOX1  IGJ  FUT7  FOXA1  GALNTL1  NRG2  FAM150B  GPR133  LRRC31  LOC100505938  FUT3  CEACAM7  LGI1  LOC389023  ABCA6  UPK1A  C1QTNF7  LOC100506758  NR3C2  LONRF2  PDK4  DES  SEMA3E  SLC27A6  ADH4  MAMDC2  IGSF10  FUT5  ANKRD20A2  CYP3A4  BTC  SRD5A2  DLK1  PART1  TFAP2B  ENPP3  PLCXD3  SH2D1B  LINC00261  PDE6A  ADH1C  C4orf19  CP  C7  FCER1A  SLC44A4  SERPINB11  LAMB4  CYP3A7  CLU  PRG4  CYP2J2  CKMT2  SELENBP1  CDO1  ACOX2  MEOX2  VSX1  SMR3B  GJC3  SOD3  KLK12  KIAA1324  TSPAN12  NDRG2  COL14A1  NCRNA00185  RNASE4  C6orf124  LOC100507311  RELN  AMOT  GPX3  GPR64  HP  MGP  AQP3  CXCL12  MUC16  FRZB  CFD  SYTL5  PLAC9  BPIFB6  SLC5A8  C5orf4  CTSG  NDNF  RIC3  ODAM  S100A1  SYT8  ITLN1  RGN  FAM189A2  FLJ13197  KRT222  SFTA2  LCN2  SLC44A3  CTTNBP2  OMD  MYO5C  SPDEF  SCARA5  PRSS36  SNED1  SFRP1  GNA14  DAGLA  ANXA9  HCG22  CH25H  MFAP4  MYZAP  CCL19  RSPH1  IL17D  EYA2  CCL23  ZNF415  MYOT  XKR4  SLURP1  CRTAC1  HPR  NUCB2  CHST6  CYTL1  CYP2B6  TTYH1  CYP2C9  SCNN1A  ZNF818P  GFAP  EYA1  CAPN6  EHF  CFHR3  NOSTRIN  SLC4A4  S100P  UGT2B7  KLHDC8A  GSTA2  ZNF285  CFH  SDPR  C13orf33  RNF183  LEPR  IL27  PZP  TMPRSS2  CEACAM1  FCGBP  PRELP  KCNJ16  RARRES1  GSTA3  LOC572558  KCNB1  AADAC  ADRA2A  TIMP4  CA3  HCAR1  PRSS27  DYNLRB2  AIF1L  VSIG10L  F13A1  MUC20  GFRA1  CCL28  SNTG2  PTPRN2  JAM2  RSPO3  LOC100289255  TMPRSS5  DNALI1  NKX3-1  ACADL  CD200R1  GALNT12  PEBP4  MANSC1  SPAG8  C9orf128  EPS8L1  PHYHIP  ZNF300P1  FXYD1  CCDC64B  CLDN8  CAB39L  ZMAT1  DHRS9  ANKRD35  MFSD6L  MUC1  PEG3  MAB21L2  LYPD2  SORCS1  TTC9  DLG2  UPK3B  FAM174B  SLITRK6  EMCN  PAX9  FGF7  GJB1  LIPH  LOC100128252  DEPTOR  ALDH3A1  GALNT5  MYH11  FGF10  ROPN1B  LOC440335  PODN  F10  KBTBD11  PTN  ITM2A  C15orf62  LOC100128164  MAMSTR  SOX10  MACC1  LOC285141  KLK11  SHISA3  SCGB1A1  ENDOU  KRBOX1  LRMP  SMPX  SLIT2  ASPA  SLITRK5  HLF  CEACAM3  ANG  F5  KIAA2022  KLF15  SPATA18  RRAGD  P2RY14  LOC100507049  C1orf168  NAAA  SYTL4  DARC  LMO7  ZNF829  SCGB1D2  DDIT4L  SERPINI2  TRNP1  TLE2  ACTG2  PSG5  TOX3  GULP1  FAM47E  PCP4L1  PLEKHA6  ROPN1  SHISA6  CYP2F1  KBTBD10  SIDT1  CLUL1  LMO3  LOC100507165  GUCA2B  ARHGAP6  LOC643037  FKSG2  DCLK1  NTN4  CAPN9  COX7A1  PMP2  TMEM45B  TMEM178  GLYATL2  WNK4  EBF1  SLC26A2  FA2H  HTR1E  KIT  PTX3  SP5  C2orf55  GDPD3  CYP1B1  SASH1  ALDH1A1  FUT6  CRLF1  CA8  AGFG2  PLEKHB1  SPAG17  BEX4  AMY1C  PAIP2B  IL1R2  CCDC110  NSUN7  TTN  ACCN1  ZBTB16  TMEM61  LOC100506689  C7orf46  ZNF471  PKHD1L1  CLIC6  SPARCL1  PHYHD1  KAT2B  LOC100506542  IL12A  WDR49  SYBU  PPL  TMEM125  PRKAA2  CCL21  C8orf84  ZNF540  PPP1R36  PPARG  ATP13A4  COBL  IKZF2  GNE  FAM55D  ZFR2  CHL1  ACPP  STK39  CNTFR  SAMD5  ZSCAN18  FLJ37644  PIK3C2G  TESC  METTL7A  NKX6-2  KRT15  FAM13C  C1orf173  DPCR1  PDGFRL  HS3ST1  DLEC1  ZNF208  NME9  PGM5  IL20RA  ZNF682  CLGN  EMP1  ARMC3  LOC644192  HOXA2  FLJ38379  BOC  TNNI2  SRPX2  MUC15  MORN5  LINC00478  B3GNT6  TSGA10  S100B  SVOPL  SLPI  SYNPO2  PTPRT  BHMT2  FAM63A  RAB17  COLEC12  PPM1L  PLN  PDZRN4  GPD1L  ENPP5  AQP1  LMO2  ZNF667  SAA3P  ZFP28  RAB40AL  IL36A  PRDM16  DMGDH  ADARB2  C18orf26  TTC18  GLYATL1  RCAN2  ANKRD37  CYP2C18  CAPS  BARX2  AOC3  SMAD9  AKAP12  SLC16A7  MEIS1  ZNF528  TSPAN19  GPIHBP1  ID4  MGLL  ANO5  LOC400891  FAM149A  ZNF880  SPNS2  IGFBP5  BMP3  HNMT  CXXC4  FGF14  CD24  ART3  FREM1  ADAMTS5  SPAG16  MUC13  MT1JP  TPPP2  LOC100507632  LOC283143  B3GNT3  PACRG  ICA1  SERPINB3  TMEM220  ECM1  TAC4  GNG4  CCDC37  C8orf47  CMAHP  BLNK  LNX1  CSN3  PIP5K1B  ELF3  PDE1A  KIAA1244  KCNE3  C1orf114  ZNF649  ARMC2  PCOLCE2  MAGI2-AS3  VSIG4  C15orf48  ZNF347  MYO1A  MYOZ1  PPARGC1A  DDAH1  ASXL3  LRRC4C  FAM198A  OR2A7  TNFRSF17  SYT13  AQP7P1  OR7A17  CPLX3  FNDC5  TIMP3  LXN  C2orf77  SLC35C1  DIO2  BCKDHB  C3orf15  CYP4F3  LOC100506795  SPATA6  C3  LIFR  PDZRN3  LOC285943  AGER  PDE2A  DMRT3  FAM153B  KRT19P2  CAPN8  ATOH8  PPAP2B  ADAMTS9-AS2  DEFB1  NLGN1  MFSD4  SERPINB13  SORT1  MUC22  SCNN1B  ABLIM3  TMEM71  MEIS2  CDH26  SHROOM3  MAP6  C15orf52  UBQLNL  SVIP  KIAA0894  GMDS  UBXN10  USP54  ZNF577  IQCD  GPT2  ZNF385D  TTTY15  NR2F1  CDH22  RORC  RNF150  SERPINB1  C1orf87  CCDC80  ELF5  C9orf24  PDE8B  CA13  LOC728763  SLC7A4  SSTR1  ZNF671  EXPH5  NR2F2  AKD1  ATP7B  RHOJ  FUT2  EMX2OS  TP53I3  TTC21A  BEND5  LAMA2  RGS17  FNDC4  GNG11  CLDN7  TMEM139  TSPYL5  ESRRG  MYH13  SPEF2  LOC100132790  SPON1  SMR3A  ATP2A3  TMEM229A  LOC283481  NFIA  COL4A4  RUNX1T1  SCN2B  ARHGAP20  CHPT1  LOC100131176  SLC5A1  KIAA1377  CHADL  PCDH9  SLAIN1  TSPAN1  CD302  ZFP2  KRT32  ENPP4  LRRC10B  SLC34A2  FP588  ADAD2  SLC37A1  CYP2C19  SMOC2  LOC100506388  PTK6  ZNF608  SERP2  SCGB2A2  TSPAN6  RBP4  PLCB4  SYNE1  EPHX2  FMO9P  RASEF  NPDC1  UBL3  GCHFR  RAI2  MIR100HG  ALDH1L1  SERPINB4  MUSTN1  MGC24103  LOC100506965  KIAA1456  RASAL1  LOC100134091  ZNF568  FBLN5  C9orf135  DHRS3  FAM184A  CCR2  TCEA3  STXBP6  TXLNG2P  C5orf27  AQP7  FAAH2  MSLN  CD34  SLC46A2  SORBS1  SLC12A2  CNN1  C7orf58  CLEC4G  CPEB3  VILL  KLRB1  NEIL1  ZNF542  GAS7  ZNF404  ACHE  ARMCX2  LOC100131581  C6  FMN1  ZNF83  SATB1  LOC284244  NFIB  RBP7  SLC13A4  ZNF135  GPRASP1  LOC145837  EDAR  TCF21  CASC1  GALNT7  EFCAB4A  CPEB1  MUC3  CPE  ZNF717  VWDE  ROPN1L  DPT  CHRNA7  SLC35F1  NR4A2  ARHGEF10L  KLHDC9  ZBTB20  CFI  CRYL1  DUSP26  SULT2B1  STK33  DOPEY2  CGREF1  AGT  LRRK2  LOC100652791  RORB  ACSM1  C1orf88  CAPS2  C1orf116  NFIX  PKIB  C18orf34  MECOM  A2ML1  DCDC5  ADAM28  LIMCH1  TC2N  RAB37  WDR69  RBPMS  FOXC1  C3orf14  CXCR2  BDH2  HMCN1  TNXB  NTRK3  KLRF1  A2M  C11orf92  ISM1  KIRREL3  TMTC2  CPN2  STAB2  LOC100507486  ZNF610  EPB41L4A  CX3CR1  TCEAL2  ZNF43  PTCHD1  HOXB2  LRG1  OR7E14P  C9orf174  EPS8L2  TNFRSF11A  LOC389033  TFCP2L1  KCNQ1  PRSS22  SAMD13  ADAMTSL3  ZNF728  DIRC1  C1QTNF3  DNAH12  FAM107B  SEPP1  RHBG  ALDH6A1  TRPV6  HOTAIRM1  C17orf97  LOC283278  SOBP  ZNF423  HTR2B  NANOG  CTNND2  ZNF345  ZNF229  STEAP4  TNNT3  LRIG1  FBP1  KCNAB1  C5orf46  FHL1  MOXD1  RNF125  PRKAR2B  LOC100507372  CCDC125  OLFML1  CYSLTR1  HS3ST2  HEPACAM2  SLITRK4  NUDT12  UTY  ADCY6  ZNF433  GRAMD3  NYX  CIDEA  ZNF91  GIMAP7  ACOX3  TMC4  ZNF429  CBR3  LRRC37A4  LOC100506123  TGFBR3  IPW  LHFP  PIK3R1  DMRTA1  GPRC5B  ZNF185  ADH1B  GPT  MEIS3P1  USP9Y  NEGR1  RGS7BP  NR2E3  SCGN  DNASE1L3  CTGF  ME3  RNF180  ZNF214  MPP7  ABLIM1  ALDH7A1  MAPT  RPS6KA6  FIGN  CD1C  PPP1R42  MLIP  GIMAP1  LOC100131138  EPHA10  TRIM2  CNRIP1  KLF8  ZNF521  LOC100507008  ITPR2  KCNK15  PDCD4  C19orf18  NNAT  SCML4  PENK  GTF2IRD2  SV2B  ZNF736  RBM47  ANKAR  TMEFF2  TLR5  NDN  LOC339524  SSBP2  SERPINB6  KCNN4  LOC388630  TFPI  GNG7  ZNF573  ZNF726  ARSF  ZNF350  RARB  C17orf110  RGS11  ASAP3  STAC2  FLT3  OLFML3  NEBL  MALL  LOC100289090  MAN1C1  LOC100292909  HSPB8  CYP3A5  DOCK3  VPS25  BEX5  LOC100509121  MMEL1  SLC16A9  PID1  CBX7  ZNF701  TPO  FMO5  CADM1  KRT23  ZMYND12  EFHA2  SHC2  FIBIN  ALDH9A1  SHC4  FYCO1  DNAH7  BSPRY  QSOX1  LDHD  SMPD3  CMA1  GPRC5D  TENC1  TCEAL7  ADHFE1  ZNF737  ARHGEF26-AS1  SELP  ZC4H2  ZC3H6  ZNF329  C8orf8  TM7SF2  RUNDC3B  LOC100507387  MUC5AC  PRRT3  SLC13A3  MIR600HG  TNFRSF10C  ANKRD42  EPB41L3  ACTA2  ETV1  SNRPN  LOC100128420  LOC219731  ZNF675  CYP2E1  FOXP1  ABCB1  MYCT1  ZNF99  SVEP1  LINC00312  ABCA10  MATN1  GIMAP8  ARL6IP5  CCDC85A  NYNRIN  C10orf90  B3GALT4  MGST2  UNC80  DIO3OS  SERPINA5  GATM  KATNAL2  GALNTL2  BNC2  CCDC42B  ZNF479  ADCY2  KRTAP4-2  PLS1  BZRAP1  APIP  GPR27  MCF2L  ZNF626  RASGRP2  LOC100509780  CA4  FAM19A2  LRRC17  FLJ36848  TSHZ2  ZNF831  SLC40A1  C5orf20  FCHO2  ZNF729  FAM162B  MUC4  WWC1  F3  SNCA  SLIT3  PLIN5  LOC100132815  KIAA1683  SPRY2  PCCA  UG0898H09  TRIM6  CELF2  TMEM170B  SAMD10  MEF2BNB-MEF2B  VASN  KLB  ABCA5  SEMA3A  TP53INP2  CLIC5  LOC728537  LOC100505806  SNURF  TTTY14  SCN3A  MACROD2  PAK3  CAST  ZNF676  RERG  HSPB2  SGCE  KIAA1370  LOC643733  TIMP2  LOC400456  CLDN23  CYB5A  ZNF730  ZNF85  LILRB4  IL17RD  GRAMD1C  NRN1  ANXA3  HMGN5  C7orf41  NOXA1  CIRBP  TEKT4P2  ZNF716  ALDH3B1  GIMAP5  GLRB  SLCO2B1  SMAGP  CGN  C15orf34  LRRC6  POLR2J2  ERN2  RMND5B  PLEKHA7  KLHDC1  C12orf26  EFEMP1  IGIP  HRCT1  CST5  TMEM129  RALGAPA2  KIAA0232  SYTL2  CXCR2P1  PDGFD  SPINK7  CDNF  IQGAP2  ZNF132  HOXB3  ZFP82  VAV3  GPRASP2  CLEC10A  ECHDC2  IGSF22  LOC641467  FBXL16  ZNF536  LOC730102  KRTAP5-8  ZNF595  RBMS3  ZNF425  C21orf88  KLK3  C22orf15  FMOD  ZNF426  SLC16A6  KL  AMICA1  LOC90499  EHD3  CCDC108  C16orf7  PIM1  ALOX15  EPHA3  CORIN  TRPC1  DDR2  KIAA1737  ST6GALNAC3  C4orf3  PRUNE2  DAAM2  CDR1  GSN  LOC100507309  C14orf45  GOLGA6C  RNASE6  RIIAD1  LOC283701  PPP1R32  C9orf95  ZNF439  ZBED3  ECSCR  ATP10B  TRIL  HOXA4  KIAA0485  TMEM232  PRINS  LINC00341  SEC31B  MEF2C  LOC100506428  HTR3B  PYGM  TNFSF12  SERPINF1  TECPR1  BCL2  ZNF583  LOC100507043  GKAP1  WBSCR17  BAIAP3  ANXA11  OCLN  GPR126  DENND2A  KBTBD3  HIPK2  TPCN1  UPK3A  ACCS  C6orf132  SHE  FAM150A  PTGER3  CYP1A1  TNFRSF13B  ST3GAL6  TNFRSF14  BHLHE22  COL4A3  FAM172A  ZNF486  LCA5L  TDRD10  TMOD1  PABPC1L2B  CITED2  DOK7  CAND2  LOC100132330  ZNF117  ZNF738  PRPH2  CPPED1  BACE2  SECISBP2L  DPYS  SLC6A4  EVPL  KRCC1  LOC100129113  SERTAD4  MYH8  O3FAR1  KIF13B  LOC154822  ANK2  BAZ2B  ADCY4  BRP44L  SLC1A1  KLHL3  CREB3L4  IL16  ELN  BMX  ACSS3  FBN1  SYNGR1  CCDC68  ZNF732  LOC389199  RADIL  C9orf103  GRID1  DUOX1  LECT1  HAND2  CYB561D1  SERPING1  ABAT  CLMN  LOC100507131  RPS6KA2  CCDC96  SEC11C  EFCAB4B  PCM1  DMWD  KCTD6  SMARCA2  UPRT  VGLL3  FRY  CPXM2  LPAR6  PPP1R12B  LOC100507780  SLC39A8  TXNIP  PPP1R1A  TMEM63A  SGSM1  LOC400043  C2orf67  FAM131C  SLC35A3  RANBP9  JAM3  WNT5B  PPFIBP2  LGI4  DUSP5  GSTM5  LDB2  CRNDE  FAM27L  C6orf123  RGMA  SPAG11B  TOB1  C11orf16  ANO8  CADPS2  PBX1  TTC22  MYO5B  TRAF3IP3  SH2D4A  KAZALD1  ADORA1  HIST1H3J  GALNT4  GOLGA4  ZNF254  PLEKHN1  TCP11L2  PHACTR2  LOC100506898  CTAGE10P  L1TD1  ARHGAP18  CTSF  FLI1  FOLR2  NXPH3  CFP  ASGR2  LMCD1  CMTM8  TMEM132B  MANEAL  C6orf192  SH3YL1  GOLPH3L  FBXL19  DACH1  PARP4  CLEC4M  SPRY1  EFNA5  TMEM40  ERVH-3  CRIP1  CLINT1  HDHD2  CCDC148  LPAR1  LOC100507053 | DPP9  ORM1  OLFML2A  IL1RAP  SLC5A4  PSME4  LOC100130920  HIST1H2AM  CCT6A  SGK1  DDX39A  SFXN1  TREML2  AP1S1  HTR7  ZNF286A  SGIP1  GPR172A  FEM1B  ZBTB20-AS1  NT5DC3  GPR84  HIST1H3F  CDK4  C9orf21  TXNRD1  TGFA  STC1  KLF11  ZNF484  TNF  PRAP1  CTSE  HOXD8  GMNN  SYCE3  HCK  AHSP  DEGS1  PCNA  SEPX1  HIST2H2AB  PAFAH1B3  PML  TTC7B  CDH15  MCAM  ITGAV  LOC341056  HIST2H2BE  KRTAP13-2  GPR63  CHST7  HIST1H2BI  PARVB  HBE1  DDX58  TMEM206  RACGAP1  SLC5A3  MYO7B  ZP3  CCT5  LRRC8C  UAP1L1  C1orf124  UBAC2-AS1  LILRA3  HSPH1  WARS  C6orf228  ATP2B1  TNFRSF4  C10orf35  XPO5  IL27RA  PLEKHG2  LAG3  F2RL1  CDR2L  STK32C  RANGAP1  HCP5  DDX60L  RFC5  CASK  MMP15  COL5A1  CCDC86  USP28  HIST1H4G  ICOS  C12orf70  HTRA4  NKX2-1  FAM176A  RGS16  ABCC4  AARSD1  FLVCR1  VMA21  CENPP  LUM  CLPB  CORO1C  SNRPB  HIST1H4A  POLD1  LOC100130776  MUSK  NKAIN1  PSMB2  LINC00346  FLVCR2  MRPS17  FLJ30403  CENPQ  PTK7  RSRC1  ELOVL3  TNFRSF8  SPATS2  ATP1B3  BORA  ITLN2  NOP2  CAV2  SLC11A1  PMAIP1  PGPEP1L  SH2D5  ATP13A3  CBX3  CCDC138  DLEU2  NDE1  IFI35  UPP1  HIST1H2BC  LILRA2  SCD  ARHGAP22  HIST1H2BM  LGALS8-AS1  TIGD3  MCM6  SAC3D1  QSOX2  MMD  MRPL36  NCS1  HIST1H2BN  NAA25  HIST1H2BD  IFITM1  CEP97  SOCS4  RNASEH2A  WDR67  ARNTL2  COL4A5  TLR2  CDKN1A  DBF4B  C4orf26  GJB4  SOCS3  TIGIT  COPZ2  KCNK13  APOL1  SLC39A14  C1orf112  FAM69A  XRCC6BP1  CENPW  NPL  AQP11  LOC440900  PNPLA1  THOC5  RFWD3  TP73  TRIM59  LAMA1  TMEM158  DNAJC6  PFKFB4  SLC18A3  CCNE2  OR1N2  KCNJ12  SLC6A9  CDH24  MCM8  ATP6V1C1  MICALL1  MRPL47  IFI30  C1orf186  ODZ3  DSG2  FKBP9L  SCO2  TAF5  TMCO2  METTL7B  EGFL6  TRMT6  MYH10  LOC100506870  CCDC77  CCNB2  OLFM2  FLAD1  CXorf57  KNTC1  MAD1L1  LOC100506831  SLED1  EMR2  RAB42  HIST1H2AD  LOC646719  ABCA1  PHF15  PLOD1  C8orf42  DVL3  DUSP2  CD300E  PARP12  MINPP1  RHEBL1  FSD1L  TRMU  FCGR3A  GALNTL4  DPP4  ADA  SNAPC1  FZD2  NEURL  STK3  TSPAN15  NPC1  GLT25D1  UNG  PRIM1  PARP14  CXCR1  RFC2  C3orf52  CDK6  SLCO1B1  HIST2H2BC  CHRM3  KIAA0895  P2RY6  CTLA4  ZC3H12A  C12orf5  DHCR7  TSPAN18  TNFRSF6B  FZD6  RRN3P2  ALDOC  CCDC24  TPH2  C1orf55  POSTN  SLC22A1  FHOD1  LOC646903  C1orf61  LGALS7  TRAIP  CNTD2  LOC100505702  FAM106CP  BID  ALG3  LOC344967  SLC44A5  FCAR  C20orf27  CHST11  HIST3H2BB  EFNB2  CATSPER1  KDM6B  CYP2S1  CHRNA6  SGPP1  NCF2  MAP4K2  BVES  GMPS  PRKAR1B  SMC4  HIST1H2BL  SOX30  GGH  ZFP64  WDR53  NAV1  PAQR3  HRG  OSBP2  ZWILCH  C5orf55  RACGAP1P  TDRKH  HIST1H2BE  COL5A2  MX2  UBE2L6  LPCAT1  PRR5L  TCP11L1  TGIF2  TUBA1C  COL13A1  AGTRAP  SRCRB4D  LOC254057  HIST2H2AA4  POC1A  JAG2  MYO5A  F2RL2  CLCN5  LOC100506469  SLC7A6  C1QTNF6  EPHB1  TMEM182  SLC1A4  NDRG4  WNT3  TNFSF11  C19orf59  ACAN  FGF1  BCL2L10  TMEM38B  ELK1  HIST1H4I  INPP4A  STX1A  TOB2P1  RECQL4  MAP7D2  CSGALNACT2  FLNA  VAX2  KPNA2  C5orf13  SLC6A10P  ITGB6  TNFSF4  GRAMD1A  CCNJL  BYSL  FAM92A1  TUBA1A  OSMR  HIST1H2BF  HIST2H4B  ASAP1  TIMELESS  HAUS8  CCDC28B  LRR1  MICAL2  ITGB4  RNF168  IFI44  C20orf72  TMCC2  ISL2  PTTG1  HCFC1  HIST1H1D  ARHGAP28  RIMS2  PSORS1C1  MSN  SIN3B  PTTG3P  LCE1F  NRM  ADAMTS2  HIST1H2BO  IGFBP3  SAMD1  HTATSF1P2  PMEPA1  ATP2B4  DGCR5  NDRG1  GIT1  APOE  DUSP7  DNAJB5  C1orf226  HKDC1  LINC00260  CLDN1  FAM106A  BOP1  EPHB4  MX1  FAM59B  NUP155  FLJ32255  SLC29A4  MOV10L1  HIST1H2BH  ELF4  ROR2  PAX6  PPPDE1  ABCC2  BDNF  SLC3A2  PQLC2  CTSL1  FAM105B  POLA2  WNT10A  RRAS2  PSMC3IP  XAF1  XPR1  CDK5R1  TTYH2  ANXA13  BICD2  POU6F2  FJX1  MEST  MGC16121  BRF2  IL32  TNS4  C6orf99  LMO1  CENPH  RELB  LYPD6B  ONECUT2  TET1  SLC30A3  C11orf84  DFNB31  POLE  FSD1  TMEM200B  AHRR  ONECUT1  GRAMD1B  COL12A1  FAM83B  CEP152  ERF  COL4A2  DUSP10  DNAH14  CBX8  LY6E  PSTPIP2  SPIRE1  AGRN  DMC1  MCM5  IDI2-AS1  ANK1  PIGX  BRIX1  C1orf51  TACC3  BRCA1  SLC15A3  BMP1  NCAPG  C4orf10  HIST1H1B  CYP26B1  POFUT1  TNN  STIP1  MICALCL  CENPV  PKP1  MGC4294  IFIT2  EIF2C2  PTTG2  LOC100128644  WNT7A  CPT1C  EFNB1  SNAI1  PLXNA1  PRIM2  TRIB3  ESPNL  WDR62  TET3  CDCA7  ZWINT  C11orf82  KIAA0040  ZNF623  KIF22  KIF20B  BRMS1  MAD2L2  SLC2A3  C14orf80  KIF15  ANKRD40  HOXB7  HIST1H2BB  C18orf54  NFE2L3  CKAP2  PKD2L1  GRM8  LOC100132006  COL24A1  CKS2  DNMT1  OR4N4  EPHB2  G0S2  WNT3A  TAP1  EPB41L4B  PDXP  C9orf140  C15orf23  ARPC1B  SMTN  MTHFD1L  HIST1H4K  SLC7A5  ACTL6A  MARK1  SOCS1  FLJ33996  ETV7  ABCD1  CHTF18  ICAM5  TMEM65  CHST1  SLC39A4  IGSF3  TGFB1  CLDN14  SEMA3C  HAPLN3  LOC144486  CDHR1  DCBLD1  HELLS  PSMB9  SNX5  PI15  FOXRED2  LETM2  MYOM3  SRSF12  SLC39A6  LOC643650  FLJ40292  SGTB  NOX4  CHML  KLK4  ABL2  GGT8P  NUDT1  CCL27  COL4A1  BAG2  PTPN14  LINC00165  TUBAL3  ERC2  LYPD6  CDK18  CCNE1  ENAH  TAF4B  KIF7  MAPRE3  DUSP9  SPRY4  GNLY  BEST2  LIM2  EPPK1  TP53TG3  SF3B4  TP63  LOC728431  SQLE  STON2  C9orf30  TYMS  CLEC7A  HIST1H4J  DLX3  SLC19A1  BCAT1  LTBP1  SKA3  TREX2  HUS1B  APOBEC3F  NEB  LOC100505592  MNX1  KIF11  SGOL2  MND1  PPP2R2C  TPCN2  SNAI2  ITGA5  KCNS3  LRRC8E  PHLDB2  RET  FAM64A  C9orf84  PPBP  COL8A1  EME1  NTSR1  NFE4  CKS1B  LRFN4  FBXO45  TTYH3  SLC16A1  LINC00162  GBP1  FADD  CDK2  MEX3B  C9orf100  FERMT1  GAD1  PBK  SLC35G1  POLE2  TBX18  UHRF1  TTL  EXT1  BCL2A1  PAQR4  CHRNA3  EGFR  CD276  SEMA4F  MB21D1  TGM2  TNFSF9  PPIF  CCNB1  ITPKA  BATF2  IGSF11  ITGA6  ANKRD29  TRPV3  PGF  ZNF282  TGFBI  GJA3  LEPREL4  PLK4  LOC339240  LRP12  HPCA  LOC440173  HMGB3  ATP8A2  HIST1H4F  CCDC150  HERC5  NRG1  MMS22L  GAP43  UBE2S  ASF1B  LOC440356  FANCB  HOMER3  KRT79  COL17A1  LOC645427  C20orf20  LRRC38  NCAPD2  CENPL  NIPA1  DFNA5  HOXD13  PPFIA1  TNFRSF10B  POLQ  MLLT11  RTKN  MCM7  FAM43A  TROAP  ZNF695  NRP2  KIAA0101  PSMD2  SMC2  ENO2  CCL8  IL12RB2  C10orf114  CELSR3  WDR54  NOS1AP  SDR9C7  NPNT  LOC100287482  SPHK1  FAM151A  HOXD9  GNA12  RBP1  NCAPH  LOC440934  RAB32  MAD2L1  DNA2  KRT9  DIAPH3  SOD2  LAPTM4B  GJC1  PRC1  ETV4  FAM111B  TYMP  WDHD1  RHOB  WNT7B  C16orf57  KIF24  TMEM81  LRP8  BMP2  C15orf27  IFNG  FANCI  CXCL6  WISP3  HOXD1  CAV1  MAGEA5  ICAM1  CCL20  NMB  MFAP3L  KIAA1524  MGC11082  CTSL2  NANOS1  TEAD2  MASTL  C1QL1  C5orf38  PLEK2  GJA1  SCN2A  TMEM194A  SUN3  GLDC  FAM71F1  CDC25A  FSTL4  STARD4  KRT6A  C21orf30  ATAD5  PANX1  CDKN3  SLMO1  CLCN2  FEN1  TNC  USP18  SPINK1  FCHO1  KIAA1804  UBE2QL1  CDCA4  ATAD2  MKI67  TNFRSF18  AFAP1L2  STAT1  C14orf33  CENPO  PPP1R1C  EPSTI1  ZNF367  RAMP1  LOC100133299  APOC2  DLGAP1  SERPINB7  S1PR5  ANO1  RMI2  PRR16  ASPHD1  LINC00256B  DPF1  RFC4  SPC24  DUSP14  VSIG1  TUBB3  RDH16  PTGFRN  PPP4R1L  NEIL3  PFN2  C1orf74  C6orf141  CXorf48  IRF6  FAM54A  SCHIP1  FCGR3B  RGS4  TMEM132A  CDCA8  E2F7  TFRC  SPC25  KRT34  EGR3  LAIR2  LOC100506670  LINC00256A  C19orf40  ARHGAP11A  HMMR  B4GALNT3  CDH3  ITGA3  APBA2  FEZ1  RPL39L  BICD1  ATP8B3  OAS3  CEP72  LOC100506507  IGFN1  MFHAS1  MCM4  RNFT2  ESCO2  TNNI3  ODC1  SPAG5  CCNF  OIP5  SYT12  SERPINH1  ACOT7  TMEM45A  SOX12  CCDC165  SCG5  SLC2A1  CDK1  KRT6C  CDCA3  SLC13A5  KIF18B  CDT1  C16orf74  TMEM97  ACVR1C  ASPM  AURKA  PKMYT1  CENPE  SGOL1  BRIP1  SLC7A11  UBE2T  GSDMA  LOC647946  CASC5  KCNMB3  LRAT  DDX60  RASGEF1A  TNNT1  NUSAP1  B3GNT4  DNMT3B  PGLYRP4  1-Dec  MTBP  MYO10  KIFC1  MB21D2  BUB1B  GABRQ  KIF20A  CD70  TEAD4  CENPM  PROCR  COL7A1  C15orf42  SKP2  PGAM5  KIF23  STIL  FADS3  ARC  MKRN3  ADAM23  CDC25B  IFI44L  FGD6  USP41  KRT33A  XCL2  BNC1  BRCA2  EIF5A2  LAMP3  OAS2  C18orf56  STAR  CDC20  TOP2A  TNIP3  CABYR  PDPN  AQP9  LOC100652886  CDYL2  RTN4R  LOC149351  ARPM1  GBP5  CENPF  BTBD16  TNFAIP3  MYBL2  CENPN  LCE3C  PAGE2  PGBD5  TMSB15A  C8orf51  MLF1  SHISA2  FAM167A  AXDND1  FAM72D  GNB5  GPNMB  PLK1  NUF2  FADS1  LAMB3  SALL4  NDC80  KLF7  C17orf53  CPA6  DLGAP5  ERCC6L  TNFAIP6  CSPG4  CARD18  IL4I1  ECT2  CCNA2  CD177  GINS2  TTK  CENPI  SNX10  DTL  DSCC1  IGF2BP3  LOC100506798  HJURP  SLC2A6  SEMA7A  B4GALNT1  DYNC1I1  DEPDC1  CD274  BIRC5  MYO1B  RBAK-LOC389458  GTSE1  LYPD1  BAI2  ABCA12  RAD51  MELK  C16orf59  FABP6  RAD54L  BUB1  FOSL1  AIM2  IFIT3  CDC6  SHCBP1  TRPM2  LOC100129781  PLAUR  IFI27  LOXL2  RBL1  GINS4  XCL1  MSC  SOX11  LCE3B  HOXA9  IL11  XRCC2  DLX5  LEMD1  FAM132A  ZIC5  MSX2P1  CLEC12B  APCDD1L  GPR172B  KIF3C  MSX2  DNAH17  GSG2  UBE2C  CDCA5  KIF4A  ANLN  CKAP2L  WFDC5  AURKB  CHRNA5  TK1  STC2  PLA2G7  MAPK12  CDC25C  CCL3  FAM89A  ALOX12P2  HIST1H2AL  APOC1  NETO2  FANCA  PTGS2  CHEK1  KIF2C  NPW  CDCA2  C12orf75  LOC401317  CTHRC1  RGS20  CDC45  RAD51AP1  WDR76  LOR  C1orf135  DLX2  MCM2  NXPH4  ESYT3  PNCK  CHST2  LOC387895  MPP4  APOBEC3B  EXO1  PLAU  WISP1  KIF18A  NUDT11  C6orf105  LAMA3  TPX2  SKA1  IL36RN  CCL7  NRIP3  MFAP2  MCM10  KRT16P3  LOC440905  CNTNAP2  KLK5  CENPA  GINS1  CREG2  E2F1  CXCL5  KRT14  FPR2  FOXM1  CCL11  PPP1R14C  SCN8A  IFIT1  COL1A1  RAG1  APOBEC3A  IGF2BP2  CEP55  ODF3L1  IL20  IL1A  IFI6  SPRR4  GDA  GUCY1B2  ALOX12B  IL1B  DEPDC1B  KIF26B  BMP8A  KREMEN2  KRT6B  LCE2B  CYP27B1  MAGEA6  BST2  MMP9  CXCL10  CCRN4L  DLX1  WDR72  ORC1  CXCL11  TCHH  TDO2  ASPRV1  OSM  CDSN  RAB3B  NEK2  TCAM1P  SLC10A4  OGDHL  ADAM12  GTSF1  LINC00491  LIPG  COL10A1  SEC14L4  GPR19  TMC7  DSG1  SHOX2  LINC00319  TNFRSF12A  TREM2  FAM40B  GAST  IL8  CYP26A1  LEPREL1  ALPK2  RASL11B  PNPLA3  TMEFF1  FOXL2  FLJ45248  EMR1  FSCN1  EN1  RSAD2  KHDC1  HOXA10  ODZ2  AFAP1-AS1  LCE3A  ARSI  SLC47A2  GAL  CLSPN  KIF14  TRIM15  KRT16P2  PPP4R4  TNFRSF9  DLL3  MMP11  ERVMER34-1  DEFB4A  NELL2  CSF3  GNGT1  NLRP7  SLC6A2  MUCL1  PPAPDC1A  MMP10  LY6K  GDPD2  WDR66  COL4A6  POTEC  KRT17  NEFL  DLX4  HSD17B6  SYT1  HMGA2  FBN2  KLHDC7B  INHBA  ISG15  PCSK9  LOC286467  KRT1  SLCO1A2  TRIP13  GPRIN1  LRRC15  LOC100507460  OASL  LAMC2  POTEE  IGFL2  CTAG1A  POTEG  PLAC1  COL11A1  HOXA11-AS1  LOC100652730  IGF2BP1  IL24  CSAG1  LCE3D  DHRS2  KRT16  C6orf10  HOXC10  POPDC3  SPP1  ZNF114  FLJ13744  WFDC12  DSC1  TM4SF19  C14orf34  HOXC8  TREM1  HOXD11  MMP13  KRTDAP  PTHLH  SERPINE1  HSPB3  GJB7  HOXD10  CSAG2  HOXB9  S100A7  SOST  POTED  DEFB103B  POTEB  CSF2  PRAME  LHX2  IL36G  CA9  HOXC9  MAGEA9  HOXC13  MAGEA4  MAGEA12  MAGEA1  MMP12  MMP1  MMP3  S100A7A  MAGEA2B  SPRR2G |
