# Supplementary material for: Prediction of the mechanism of miRNAs in laryngeal squamous cell carcinoma based on the miRNA-mRNA regulatory network
Source: PeerJ. 2021 Aug 24;9:e12075. doi: 10.7717/peerj.12075 (PMC8395572; doi:10.7717/peerj.12075)
Supplement: Supplemental Information 9 [file peerj-09-12075-s009.docx]

Table S3 miRNAs and mRNAs in miRNA-mRNA regulatory network.

| **miRNAs** | **mRNAs** |
| --- | --- |
| miR-338-3p  miR-362-3p  miR-582-5p  miR-140-5p  miR-590-5p  miR-532-5p  miR-455-5p  miR-455-3p  miR-140-3p  miR-199b-5p | ARPC1B  COL1A1  ICOS  MTHFD1L  SLC35G1  TNFSF11  CHRM3  DUSP10  E2F1  TGFBI  AP1S1  CBX3  DFNA5  DLX5  FBXO45  FZD2  MARK1  MASTL  MEX3B  RASL11B  SCHIP1  SLC16A1  TTK  BAG2  BMP2  ERC2  FEN1  GIT1  LHX2  LRAT  MINPP1  MMD  MYO10  NPL  PFN2  SLC2A1  STC2  STK3  TTYH2  RSAD2  SKP2  ZNF367  ERCC6L  GBP1  TAP1  CENPP  GJC1  MFAP3L  POU6F2  SOCS3  SOX11  ACAN  CLCN2  COL4A5  FAM69A  IGF2BP2  KLF7  PAX6  PNCK  SLC39A14  SOX12  TIGIT  TMEFF1  TSPAN18  ABCA1  ADA  AHRR  ATP2B1  CD274  CHRNA5  COL4A1  EIF5A2  HELLS  HOXA9  HSPH1  ITGA6  LTBP1  PAQR4  SGIP1  SIN3B  SLC10A4  SLC30A3  SLC6A9  TIGD3  AQP11  DEPDC1B  DUSP14  FSTL4  FZD6  GPR63  ITGA3  KIAA0040  PARP12  PDPN  SNAI1  UNG |
